# DBA/2J Mice Are Susceptible to Diabetic Nephropathy and Diabetic Exacerbation of IOP Elevation

**DOI:** 10.1371/journal.pone.0107291

**Published:** 2014-09-10

**Authors:** Ileana Soto, Gareth R. Howell, Cai W. John, Joseph L. Kief, Richard T. Libby, Simon W. M. John

**Affiliations:** 1 The Jackson Laboratory, Bar Harbor, Maine, United States of America; 2 The Howard Hughes Medical Institute, Bar Harbor, Maine, United States of America; 3 Departments of Ophthalmology and Biomedical Genetics, University of Rochester Medical Center, Rochester, New York, United States of America; 4 Department of Ophthalmology, Tufts University of Medicine, Boston, Massachusetts, United States of America; Instituto Murciano de Investigación Biosanitaria-Virgen de la Arrixaca, Spain

## Abstract

Some pathological manifestations of diabetes in the eye include retinopathy, cataracts and elevated intraocular pressure (IOP). Loss of retinal ganglion cells (RGCs) in non-proliferative stages of diabetic retinopathy and small increases in IOP in diabetic patients has raised the possibility that diabetes affects the development and progression of ocular hypertension and glaucoma. The *Ins2^Akita^* mutation is known to cause diabetes and retinopathy on a C57BL/6J (B6) background by as early as 3 months of age. Here, the impact of the Akita mutation on glaucoma was assessed using DBA/2J (D2) mice, a widely used mouse model of ocular hypertension induced glaucoma. In D2.*Ins2^Akita/+^* mice, the contribution of diabetes to vascular permeability, IOP elevation, RGC loss, and glaucoma development was assessed. D2.*Ins2^Akita/+^* mice developed a severe diabetic nephropathy and early mortality between 6–8 months of age. This agrees with previous reports showing that the D2 background is more susceptible to diabetes than the B6 background. In addition, D2.*Ins2^Akita/+^* mice had vascular leakage, astrocyte reactivity and a significant increase in IOP. However no RGC loss and no anterograde axonal transport dysfunction were found at 8.5 months of age. Therefore, our data show that despite severe diabetes and an increased IOP compared to controls, RGCs do not lose axon transport or degenerate. This may be due to a DBA/2J-specific genetic modifier(s) that could provide novel and important avenues for developing new therapies for diabetic retinopathy and possibly glaucoma.

## Introduction

Diabetes is the leading cause of blindness among adults between 20 to 74 years old in the United States [1], [2]. Diabetic retinopathy is characterized by vascular changes that lead to disruption of the blood retinal barrier, microaneurysms, hemorrhages, and late onset vascular proliferation [3], [4]. Neuronal cell death at non-proliferative stages of diabetic retinopathy has also been observed in human disease and experimental models [5], [6], [7], [8]. Glaucoma is characterized by the loss of retinal ganglion cell (RGCs) and elevated intraocular pressure (IOP) is a major risk factor. In addition, diabetic patients have higher IOP levels (+2 to 3 mmHg) than non-diabetic people [9], [10], [11], [12]. While this increase in IOP is small, it has been suggested that the additional elevation in IOP could increase the stress on RGCs and consequently the risk of glaucomatous RGC loss [13].

Due to inconsistencies among epidemiological studies no clear association of diabetes with glaucoma has been established [13]. In fact, it has been suggested that early stages of diabetes may have neuroprotective effects on RGCs in glaucoma [14], [15]. Similarly, two experimental studies inducing IOP elevation in diabetic rats reported contradictory results in terms of diabetes effects on the rate of progression of glaucomatous neurodegeneration [16], [17]. One study reported that short-term hyperglycemia, a feature of diabetes, delayed axonal degeneration and RGC loss in a rat experimental model of glaucoma [15]. A second study, also in a rat experimental model, found that hyperglycemia increases apoptosis in RGCs after induced IOP elevation [16]. It is still unclear if diabetes affects glaucoma development or rate of progression, or if any effects of diabetes on glaucoma vary with genetic constitution.

Mouse models for diabetes have shown some pathological similarities to human diabetic retinopathy that include vascular leakage, RGC loss, and glial changes [6], [18], [19], [20]. Akita mice have a single nucleotide substitution in the Insulin II gene (*Ins2^Akita^*) that causes Type I diabetes. On the C57BL/6J (B6) genetic background, the Akita mutation causes protein misfolding, degeneration of the pancreatic ß cells, and consequently hyperglycemia [21]. B6.*Ins2^Akita^* mice also develop features of diabetic retinopathy including loss of RGCs [6], [18]. DBA/2J mice, a commonly used genetic model of glaucoma, develop ocular hypertension (IOP elevation) that ultimately leads to the death of RGCs and optic nerve degeneration [22], [23], [24]. To study the role of genetic background on diabetes development and progression, the *Ins2^Akita^* mutation has been backcrossed into the DBA/2J strain (D2.*Ins2^Akita/+^* [25]). D2.*Ins2^Akita/+^* mice develop diabetes that is comparable to B6.*Ins2^Akita/+^* mice, but they develop a more severe diabetic nephropathy. Here we use D2.*Ins2^Akita/+^* mice to determine the contribution of diabetes to diabetic retinopathy, IOP elevation, and RGC loss in this genetic context.

## Materials and Methods

### Ethics Statement

The Jackson Laboratory animal care and use committee approved the research presented in this manuscript. All experiments were performed in compliance with the ARVO statement for use of animals in ophthalmic and vision research.

### Mouse strain and husbandry

All mice were housed in a 14-hour-light/10-hour-dark cycle under previously described conditions [26]. Mice heterozygous for the *Ins2^Akita^* mutation on the DBA/2J genetic background (D2.*Ins2^Akita/+^*) were obtained from the Jackson Laboratory (Stock Number 007562). To produce experimental animals D2.*Ins2^Akita/+^* mice were bred to DBA/2J mice. Siblings homozygous wild-type for the *Ins2* gene (D2.*Ins2^+/+^*) were used as controls and housed with their diabetic littermates under the same conditions in the same animal facility to minimize environmental differences. A recent study has demonstrated that estrogen may protect female Akita mice against diabetes [27]. Because hyperglycemia in D2.*Ins2^Akita/+^* female mice is limited and variable, only male mice were used for the study. Experiments were performed in male mice at 6 and 8.5 months old.

### Assessment of diabetes-relevant phenotypes and survival

To determine *blood glucose levels*, the AlphaTRAK blood glucose monitoring system (Abbott) was used. A drop of blood after tail puncture was used for the test in 10 mice per genotype and age. Mice health condition was monitored weekly and body weight for all mice was measured monthly from wean age. This measurement offered an additional measure of disease progression and also allowed monitoring of the general health of the mice. The inability of a mouse to feed itself was established as a uniform humane end point for all mice. DietGel Recovery (Clear H_2_O) barrier packs were provided in addition to the pellet food to mice that were reaching the established end point. When mice reached their end point, they were euthanized via cervical dislocation or by cardiac perfusion after anesthesia.

For the *FITC-dextran amine vascular permeability assay* 5 mice (10 retinas) per genotype were injected in the tail vein with FITC-dextran amine 70 KDa (FDA 100 µg/g; Invitrogen). After 30 min, mice were euthanized and whole eyes were fixed in 4% paraformaldehyde overnight (ON) at 4°C. Whole retinas were dissected and incubated with peroxidase (POD)-conjugated anti-FITC (1∶1000, Roche) and FITC tyramide amplification (TSA, Perkin Elmer) was used for detection and amplification of the FITC signal in the retina. After tyramide amplification, retinas were incubated with GFAP antibody (Dako; 1∶500) for four nights at 4°C, followed by incubation with secondary antibody for 2 hrs. Retinas were mounted with Aqua Poly/Mount. For quantitative analysis of GFAP staining in the retina, 2 pictures were taken per each of the 4 retinal quadrants in the retina periphery. GFAP fluorescence labeling was analyzed with the imaging software Imaris (Bitplane Scientific Software).

For the assessment of *diabetic nephropathy*, following cardiac perfusion and post-fixation with 4% paraformaldehyde, kidneys were dissected free, washed in PBS, weighed, and cryoprotected with 30% sucrose ON at 4°C. 12 µm cryosections were incubated ON at 4°C with the following primary antibodies: Nestin (1∶200, Chemicon); CD31 (PECAM1; 1∶100, BD Pharmigen); MECA79 (1∶50, Santa Cruz Biotechnology); and CD45 (1∶100, BD Pharmigen). After PBT washes (PBS, 0.5% Triton-X100) sections were incubated in Alexa-conjugated secondary antibodies (1∶1000, Invitrogen) for 2 hrs, incubated with DAPI and mounted with Aqua Poly/Mount.

### Assessment of glaucoma-relevant phenotypes

*Slit-lamp bio-microscopy* was used to track iris disease in all mice and *IOP measurements* were performed as previously described [28], [29], in 10 mice per genotype (20 eyes) at 6 months and 19 mice per genotype (38 eyes) at 8.5 months of age. IOP elevation in D2 mice is secondary to a depigmenting iris disease [30]. Slit lamp examination at age 6 and 8.5 months in 10 mice per genotype and age detected no differences in the iris disease of the diabetic D2.*Ins2^Akita/+^* mice when compared with D2.*Ins2^+/+^* mice. *Axon counts* were performed on retro-orbital optic nerve sections stained with paraphenylenediamine (PPD) as previously described [23], [31]. Briefly, using the software ImageJ 1.48, axonal counts were performed by converting 20 images per optic nerve in one stack of images, cropping the stack of images and counting axons manually. Using Metamorph software, the total area counted as well as the total optic nerve area were tracked and used for the final count computation. The total counted area from the optic nerve was >10% and the final count was calculated and expressed as number of axons per optic nerve [22], [23]. Ages and sample sizes are detailed in the results and figure legends. A one-way ANOVA was used for statistical analysis.

*Axon transport analysis* was performed on 4 (8 colliculi) 8.5-month-old mice. Alexa Fluor 488-Cholera Toxin subunit B (CTB) conjugate (Invitrogen) was injected into the vitreous as described previously [32]. After 72 hours, mice were anesthetized and euthanized via 4% paraformaldehyde cardiac perfusion. After perfusion and submersed fixation with 4% paraformaldehyde, brains were cryosectioned at 50 µm. Alexa Fluor 488 was visualized using a fluorescence microscope (Zeiss). For image quantification 8 pictures of each superior colliculus were taken and intensity statistics were generated after surface segmentation using the same threshold criteria (Imaris, Bitplane Scientific Software).

*RGC quantification.* Flat-mounted retinas were fixed in buffered 4% PFA ON at 4°C with gentle rocking, washed with PBT and incubated with rabbit polyclonal antibodies against tubulin ß-III (TUBB3; 1∶500, Sigma) and monoclonal GFAP (1∶500, DakoCytomation) for four nights at 4°C. After PBT washes, the retinas were incubated in the respective Alexa-conjugated secondary antibodies (1∶1000, Invitrogen) for 2 hrs. The retinas were washed in PBT and mounted in Aqua Poly/Mount (Polysciences). For quantification of TUBB3+ RGCs, four cuts were made in whole retinas to divided in 4 equal quadrants and two digital images (350 µm×350 µm) were taken in the peripheral retina in each quadrant. Manual counts were performed using the cell counter plugin from the ImageJ 1.48 software. Number of retinas per group has been included in the results and figure legends.

### Statistical Analysis

Statistical analysis was performed using a commercially available statistical software package (JMP, version 10.0, USA). The results were expressed as mean ± SD. Statistical differences were assessed using one-way analysis of variance (ANOVA); p<0.05 was considered statistically significant. Experiments were performed at least three times.

## Results

### D2.*Ins2^Akita/+^* die prematurely, likely due to severe kidney dysfunction

**Figure 1:**
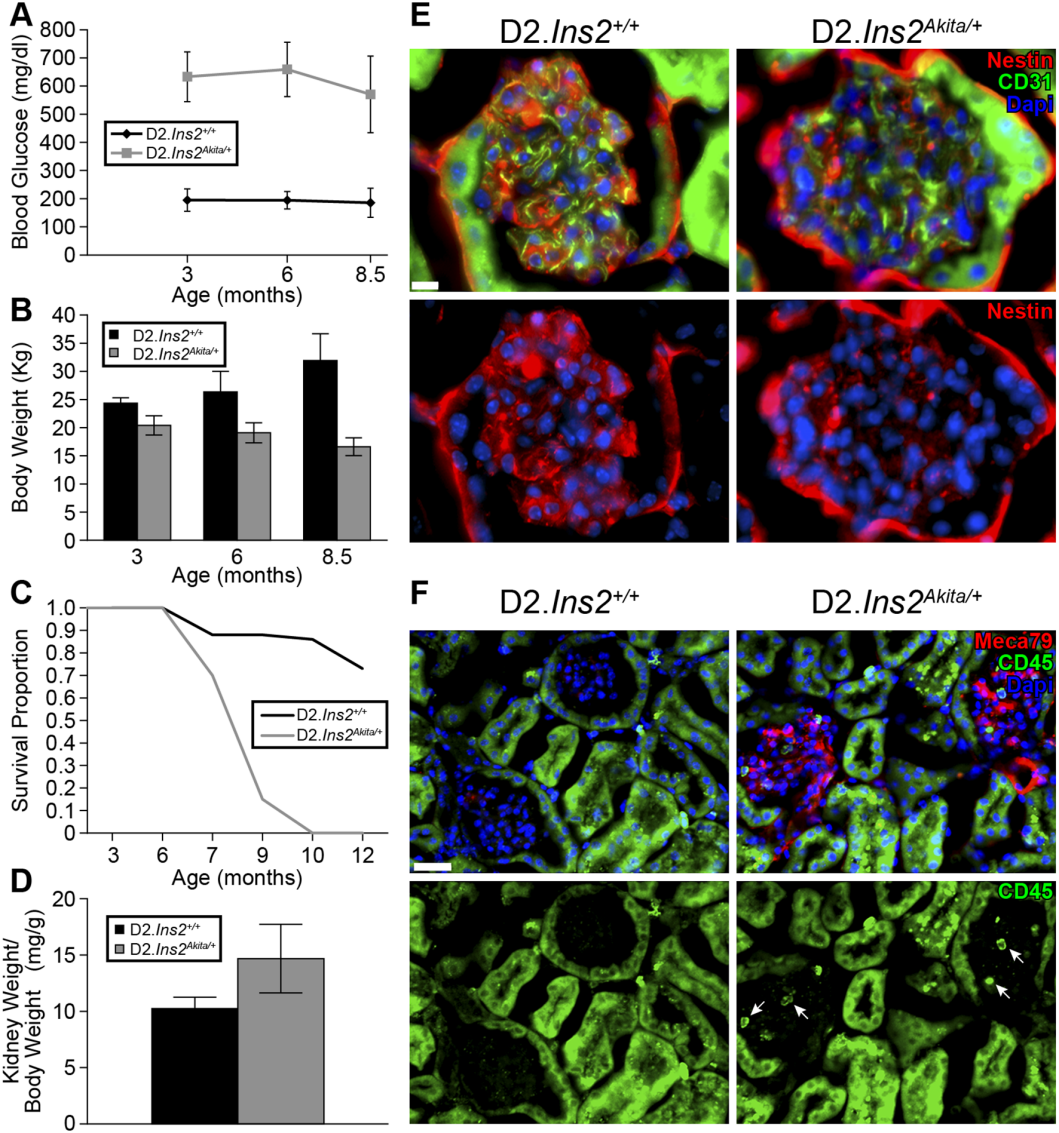
Severe kidney dysfunction and premature death in D2.*Ins2^Akita/+^* mice. (A) High and sustained blood glucose levels were found in males D2.*Ins2^Akita/+^* mice compared to their respective D2.*Ins2^+/+^* littermates during the time of the study (n = 10 mice for each genotype and age; *p*<0.001 for each age). (B) Severe loss of weight and wasting were found in D2.*Ins2^Akita/+^* compared with D2.*Ins2^+/+^* mice (n = 10 for each genotype and age; *p*<0.001 each age). (C) Sudden and early death occurred in the diabetic D2.*Ins2^Akita/+^* before 9 months (n>40 mice). (D) Kidney weight/body weight ratio was significantly higher in D2.*Ins2^Akita/+^* mice versus D2.*Ins2^+/+^* (n = 10 kidneys). (E–F) Pathological changes associated with diabetic kidney disease at 6.5 months. (E) Decreased immunoreactivity of nestin was evident in D2.*Ins2^Akita/+^* glomerulus when compared with D2.*Ins2^+/+^*. No differences in CD31 immunoreactivity in the glomerular capillary loop were found between diabetic and wild-type mice. (F) MECA79 immunoreactivity was observed in the glomeruli of D2.*Ins2^Akita/+^* mice, but not in D2.*Ins2^+/+^* glomeruli. The MECA79 immunoreactivity was coincident with CD45 positive leukocytes (arrows) in these affected glomeruli. Non-specific green fluorescence in kidney tubules was observed. Scale bars: (E) 10 µm, (F) 30 µm.

To assess the impact of DBA/2J (D2) genetic background on diabetes-induced IOP elevation and RGC loss, cohorts of male D2.*Ins2^Akita/+^* and D2.*Ins2^+/+^* control mice were established and aged. Each cohort contained at least 40 mice. For each mouse, the levels of glucose in the blood were measured at 3, 6 and 8.5 months of age. Consistent with previous reports [25], D2.*Ins2^Akita/+^* mice had significantly higher blood glucose levels than D2.*Ins2^+/+^* control mice (Fig. 1A; n = 10; *p*<0.001). However, at 6 and 8 months of age, D2.*Ins2^Akita/+^* mice had significant weight loss compared D2.*Ins2^+/+^* controls (Fig. 1B; n = 10 for each genotype; *p<*0.001 for each age). The significant weight loss was followed by premature death of D2.*Ins2^Akita/+^* mice by 9 months of age, with only 15% surviving, compared to 88% of D2.*Ins2^+/+^* control mice (Fig. 1C; n = 40 for each genotype; *p*<0.001).

Given that D2.*Ins2^Akita/+^* mice have severe albuminuria and kidney dysfunction by 6 months of age [25], the development of severe diabetic nephropathy and pyelonephritis have been proposed to be contributors of the increased mortality in D2.*Ins2^Akita/+^* [33] but never fully determined. Therefore, the kidneys of D2.*Ins2^+/+^* and D2.*Ins2^Akita/+^* mice were examined. Significant kidney hypertrophy (determined by kidney weight/body weight) was observed in D2.*Ins2^Akita/+^* mice at 6 months of age compared with aged-matched control mice (Fig. 1D; n = 10 for each genotype; *p*<0.001). At 6.5 months of age, immunoreactivity of nestin (a marker of podocytes in glomeruli) was greatly reduced in the glomeruli of D2.*Ins2^Akita/+^* mice compared to D2.*Ins2^+/+^* mice (Fig. 1E). Podocytes are essential for the function of the kidney and so this confirms significant kidney dysfunction in D2.*Ins2^Akita/+^* mice. We also assessed inflammation as a second marker of kidney dysfunction using immunoreactivity for MECA79, an antibody that binds activated L-selectin ligands. MECA79 was detected in the glomerular capillary loops of kidneys from D2.*Ins2^Akita/+^* mice and not present in D2.*Ins2^+/+^* control mice (Fig. 1F). The activation of L-selectin coincided with the infiltration of CD45 positive leukocytes in the diabetic glomeruli (Fig. 1F) suggesting a chronic inflammatory response in the kidneys of D2.*Ins2^Akita/+^* mice that was not observed in D2.*Ins2^+/+^*. Therefore, our data add further evidence that severe kidney dysfunction is a major cause of premature death in D2.*Ins2^Akita/+^* mice.

### D2.*Ins2^Akita/+^* mice show early stages of diabetic retinopathy

**Figure 2:**
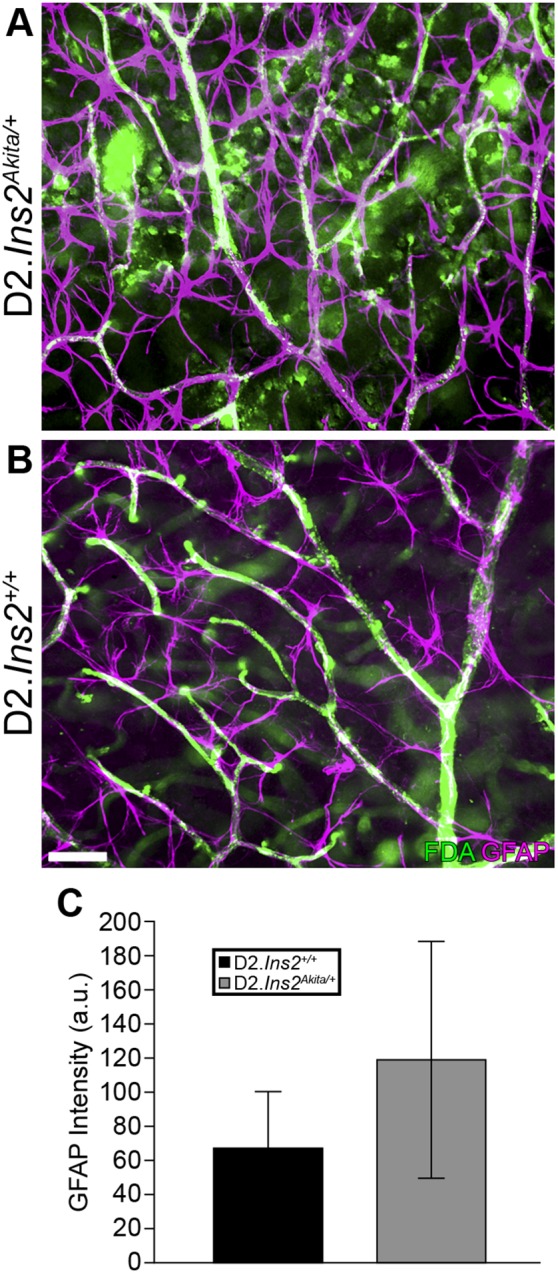
Vascular leakage and astrocytes reactivity in D2.*Ins2^Akita/+^* retinas. (A, B) To determine if the Akita mutation caused an increase in vascular permeability in DBA/2J mice, systemic injections of FITC-dextran amine 70 kDa were made into 6 month old D2.*Ins2^+/+^* and D2.*Ins2^Akita/+^* mice. Astrocytes were labeled with GFAP (purple). While FITC fluorescence signal was restricted to blood vessels in D2.*Ins2^+/+^* retinas (n = 10), there was clear leaked fluorescence signal in the majority of D2.*Ins2^Akita/+^* retinas (8 out of 10 retinas examined). (C) GFAP fluorescence intensity (purple) was significantly increased in D2.*Ins2^Akita/+^* retinal astrocytes when compared with D2.*Ins2^+/+^* retinas (*p<*0.05, n = 10). Scale bar: 50 µm.

To assess the impact of diabetes on retinal phenotypes in D2.*Ins2^Akita/+^* mice, retinal vascular permeability and reactive gliosis, two known pathological and early hallmarks of diabetic retinopathy [34] were assessed. Previously, it was shown that blood-retinal barrier (BRB) breakdown and reactive gliosis occur in B6.*Ins2^Akita/+^* mice and that these changes preceded RGC loss and neuroinflammation [6], [18], [35]. To determine the impact of DBA/2J genetic background on BRB breakdown and reactive gliosis, retinas from 6 months old D2.*Ins2^Akita/+^* and D2.*Ins2^+/+^* mice were examined after systemic injection of FITC-dextran amine 70 kDa. In D2.*Ins2^Akita/+^* mice, fluorescence signal was observed leaking from vessels and into surrounding regions and cells (Fig. 2A). Leakage of fluorescent tracer was observed in 80% (8 out of 10 eyes) of the flat-mounted retinas from D2.*Ins2^Akita/+^* mice. Leakage of the fluorescent tracer was never observed in retinas from D2.*Ins2^+/+^* mice (Fig. 2B; n = 10 retinas for each genotype). To assess reactive gliosis, levels of glial fibrillary acidic protein (GFAP), an astrocyte- and Müller glial cell-specific intermediate filament protein that is commonly upregulated in disease, was assessed. Expression of GFAP was significantly increased in retinal astrocytes and Müller glia surrounding leaking vessels in retinas from D2.*Ins2^Akita/+^* mice compared to D2 controls (Fig. 2B–C). Together these data show that by six months of age that the D2.*Ins2^Akita/+^* mice have clear signs of early stages of diabetic retinopathy.

### Diabetes exacerbates IOP elevation in D2.*Ins2^Akita/+^* mice

**Figure 3:**
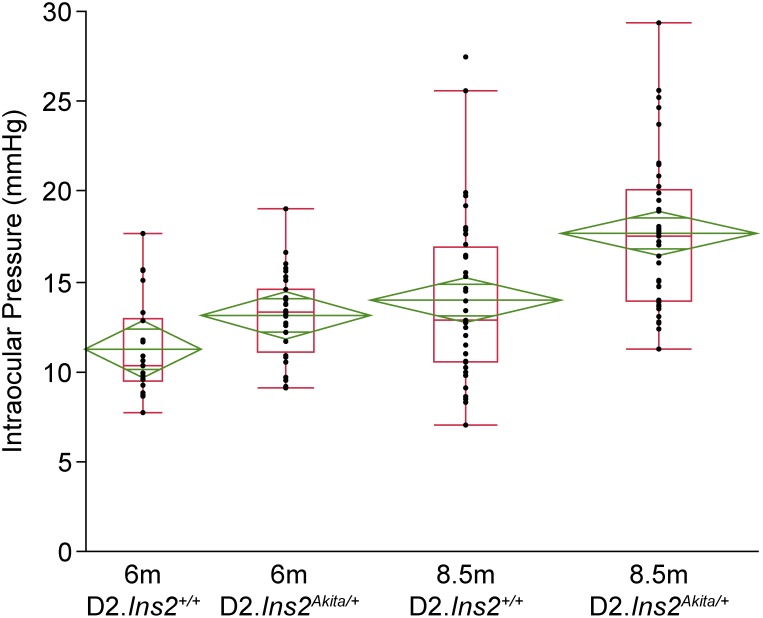
The Akita mutation increased IOP in DBA/2J mice. To determine if the Akita mutation caused an increase in IOP in DBA/2J mice, IOP were recorded from D2.*Ins2^+/+^* and D2.*Ins2^Akita/+^* mice at 6 and 8.5 months old. No significant differences in IOP measurements were found between diabetic D2.*Ins2^Akita/+^* and D2.*Ins2^+/+^* at 6 months of age (n = 20 eyes for each genotype; *p* = 0.106). However by 8.5 months diabetic D2.*Ins2^Akita/+^* showed significant higher IOP levels when compared with wild type D2.*Ins2^+/+^* (n = 38 eyes for each genotype; *p*<0.001).

Hyperglycemia is associated with elevation of IOP in patients with diabetes [36] and IOP is a major risk factor for glaucoma. Male DBA/2J mice develop IOP elevation between 7 and 9 months of age [23], [24]. Therefore, the impact of diabetes on IOP elevation was determined using D2.*Ins2^Akita/+^* mice. IOP measurements were made at 6 months of age (prior to IOP elevation in male D2 mice, only males used in study see Methods) and 8.5 months of age (during IOP elevation). No significant difference in IOP elevation was found between D2.*Ins2^Akita/+^* (13.22±2.47) and D2.*Ins^+/+^* (11.62±3.57) mice at 6 months of age (Fig. 3). However, IOP levels were significantly higher in the D2.*Ins2^Akita/+^* (17.60±4.30) mice at 8.5 months compared to D2.*Ins^+/+^* (13.90±4.66) mice (Fig. 3, *p<*0.001). This indicates that diabetes phenotypes exacerbate IOP elevation in DBA/2J mice.

### D2.*Ins2^Akita/+^* mice do not show RGC dysfunction and loss

**Figure 4:**
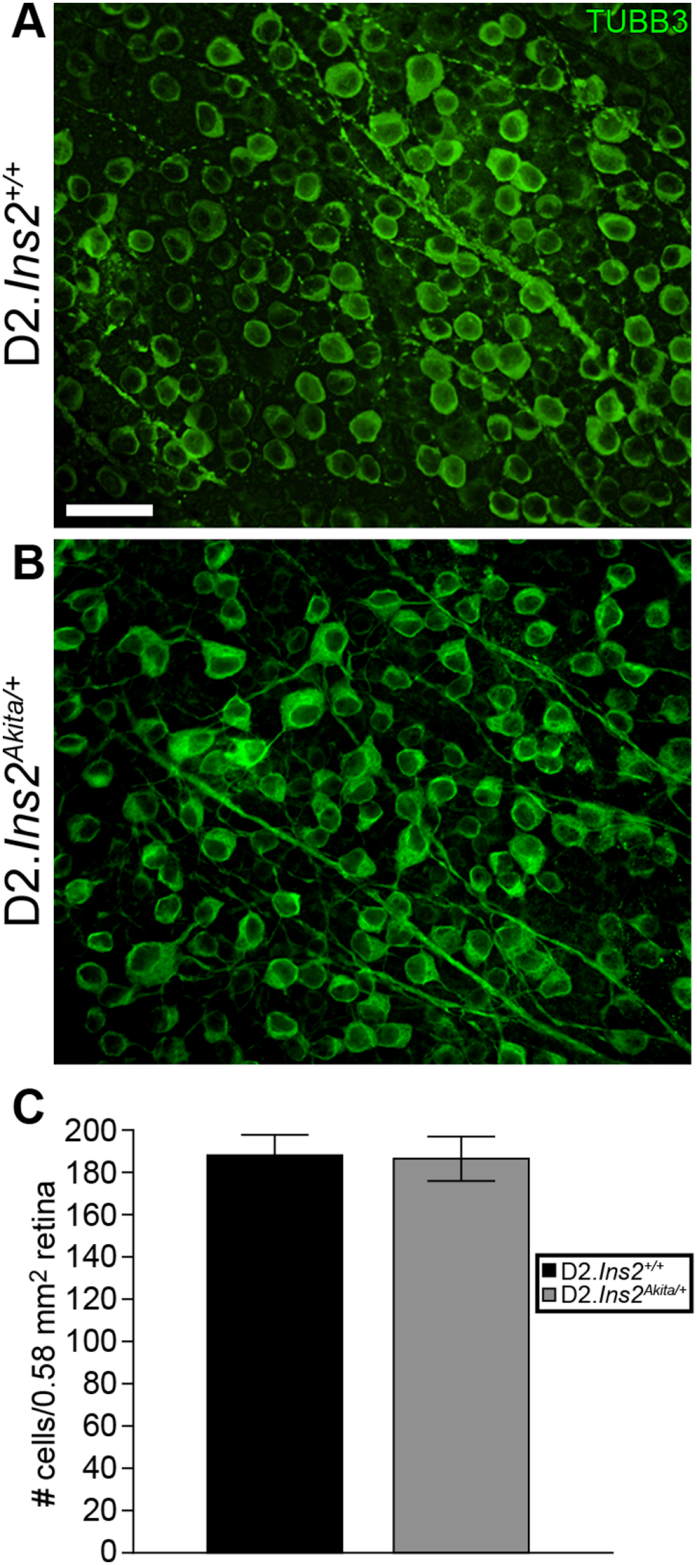
The Akita mutation does not cause RGC death out to 8.5 months of age in DBA/2J mice. (A, B) To determine if the Akita mutation leads to RGC loss in DBA/2J mice, retinas from 8.5 month old D2.*Ins2^Akita/+^* and D2.*Ins2^+/+^* mice were stained for the RGC marker TUBB3+. (C) TUBB3+ cell counts showed that there was no significant difference in RGCs detected in retinas of D2.*Ins2^Akita/+^* mice compared with D2.*Ins2^+/+^* mice (n = 8 retinas for each genotype; *p* = 0.138). Scale bar: 30 µm.

On the B6 genetic background the *Ins2^Akita/+^* mutation causes significant RGC loss by 3 months of age in some studies [6], [18] and by 6 months in others [37]. The vast majority of RGC loss due to IOP elevation occurs in DBA/2J mice between 10–12 months of age [23]. Since D2.*Ins2^Akita/+^* are not generally viable past 9 months of age (Fig. 1), RGC loss was assessed in D2.*Ins2^Akita/+^* mice at 8.5 months using an established method of counting TUBB3+ RGCs [38]. At this age, based on RGC loss at 3 to 6 months of age in B6.*Ins2^Akita/+^* mice, which have less severe diabetes [6], there should be a long enough exposure to diabetes phenotypes to kill RGCs. Furthermore, the additional IOP insult in the D2 background, which is greater in D2.*Ins2^Akita/+^* mice, would be expected to exacerbate the demise of RGCs [23], [24]. Despite this, there was no detectable loss of RGCs in D2.*Ins2^Akita/+^* mice at 8.5 months of age (Fig. 4).

**Figure 5:**
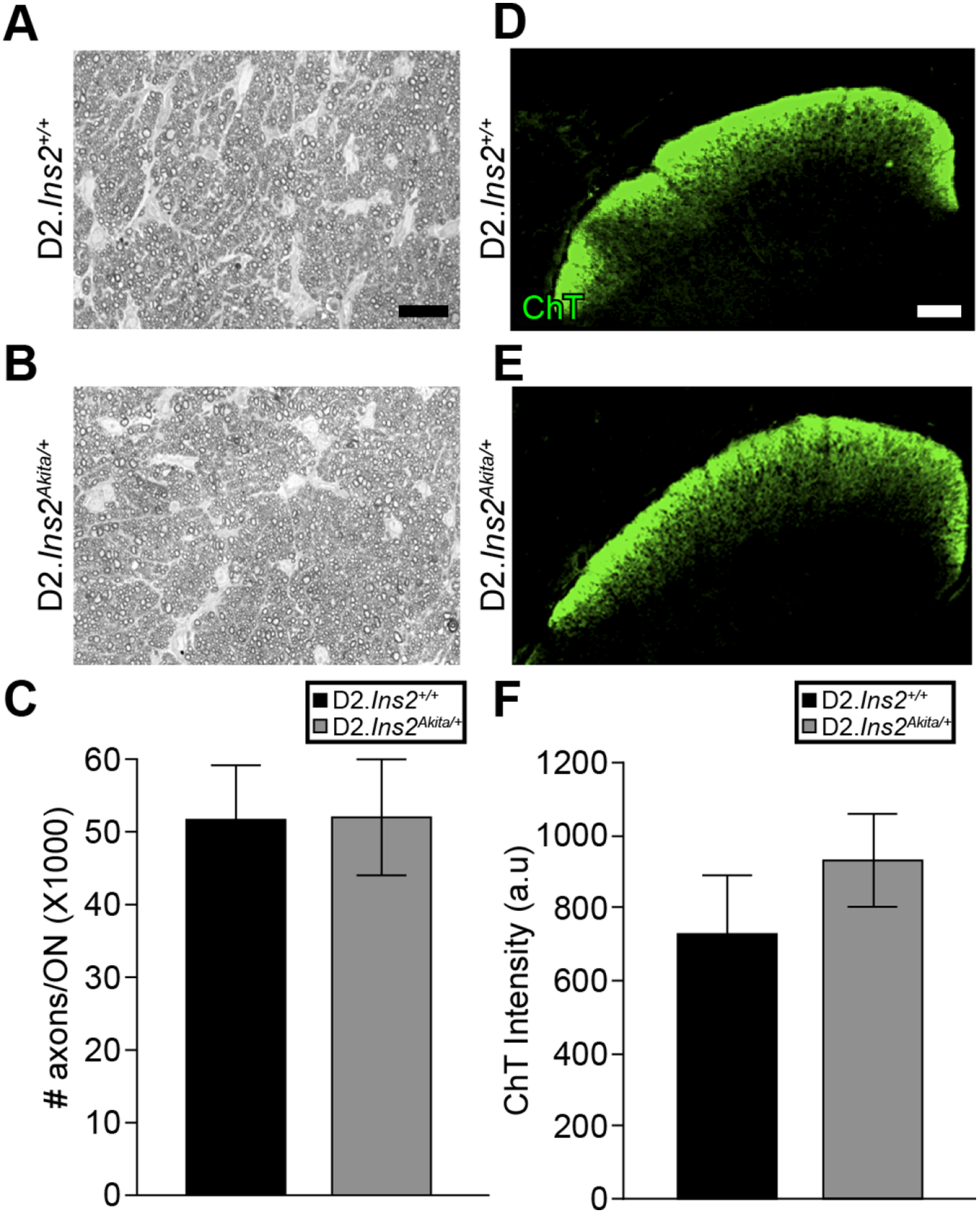
The Akita mutation does not result in early axon degeneration or axon transport deficiency in DBA/2J mice. Optic nerve degeneration is a hallmark of glaucoma and optic neuropathies. To determine if the Akita mutation leads to RGC axon loss and optic nerve degeneration in DBA/2J mice, optic nerves from 8.5 month old D2.*Ins2^Akita/+^* and D2.*Ins2^+/+^* mice was assessed using PPD staining (A, B). All of the optic nerves examined from both diabetic and wild-type mice were categorized as having mild damage, which means there was no detectable (or only a low level of damage) damage that is consistent with aged and genetically matched wild-type mice that do not develop glaucoma or diabetic retinopathy. (C) Quantitative analysis of axon counts in 8.5 months old optic nerves showed no differences between D2.*Ins2^+/+^* and D2.*Ins2^Akita/+^* (n = 20 optic nerves for each genotype; p = 0.894). (D–F) Loss of axon transport is a sensitive and early sign of a glaucomatous insult to RGCs and/or RGC dysfunction. To determine if the Akita mutation leads to disruptions in RGC axon transport in DBA/2J mice, intravitreal injections of a fluorescently labeled tracer, CTB-488, were made into 8.5 month old D2.*Ins2^Akita/+^* and D2.*Ins2^+/+^* eyes. (D, E) the superior colliculi of 8.5 months old D2.*Ins2^Akita/+^* mice appeared to fill the same as age-matched D2.*Ins2^+/+^* mice. (F) Quantification of the fluorescent intensity showed there was no significant difference between D2.*Ins2^+/+^* and D2.*Ins2^Akita/+^* mice (n = 8 colliculi for each genotype; *p* = 0.428). Scale bar: (A, B) 50 µm, (D, E) 150 µm.

To determine whether RGC axon integrity and function was diminished in D2.*Ins2^Akita/+^* mice, a well-established optic nerve damage assessment protocol and an anterograde axon transport assessment were used (see Methods). For optic nerve assessment, optic nerve sections were stained using paraphenylenediamine (PPD) that differentially stains sick and dying axons [23], [31]. PPD stained nerves were analyzed using a validated system for assessing optic neuropathy [23], [31]. PPD staining can also detect damage to single axons. Thus, it can also reveal subtle differences in RGC axon damage between D2.*Ins2^Akita/+^* and D2.*Ins2^+/+^* mice. Consistent with the data analyzing RGC somas (see above), there was no optic nerve damage present in either D2.*Ins2^Akita/+^* or D2.*Ins2^+/+^* mice (Fig. 5A–C). This observation was confirmed by axon counting (Fig. 5C). Anterograde axonal transport dysfunction is a measure of RGC function and occurs prior to RGC axon degeneration in glaucoma [39]. Anterograde axonal transport was examined in the superior colliculus of D2.*Ins2^Akita/+^* and D2.*Ins2^+/+^* mice using the fluorescent tracer Alexa Fluor 488-CTB. No significant differences were observed between D2.*Ins2^Akita/+^* or D2.*Ins2^+/+^* mice indicating that, by this measure, RGC axons appear to be functioning normally (Fig. 5D–F).

## Discussion

The DBA/2J inbred mouse strain is widely used to study a variety of human diseases particularly ocular hypertension and glaucoma. DBA/2J mice have also been shown to be susceptible to kidney dysfunction and disease [25], [40]. As diabetes has been suggested to be a risk factor for IOP elevation, glaucoma and kidney disease we used male mice from the DBA/2J.*Ins2^Akita/+^* strain to assess the impact of diabetes on retinal and optic nerve phenotypes and kidney dysfunction. Our data show that D2.*Ins2^Akita/+^* mice die prematurely and this is likely due to a severe kidney dysfunction, more severe than that observed in B6.*Ins2^Akita/+^*mice. This confirms previous findings that the DBA/2J genetic background harbors genetic loci that predispose DBA/2J mice to kidney dysfunction. Several quantitative trait loci have been identified previously [40] but the underlying genetic variations remain unknown. The severity of the kidney phenotype in DBA/2J.*Ins2^Akita/+^* mice makes this strain an attractive model for identifying specific genetic variations that underlie susceptibility to diabetic nephropathy.

Analysis of kidneys from male D2.*Ins2^Akita/+^* mice showed a significant renal hypertrophy that was accompanied by a decrease in glomerular-specific nestin immunoreactivity at 6.5 months of age. It has been shown that downregulation of nestin by podocytes in the glomerulus [41], [42], is correlated with apoptosis induction in podocytes during diabetes-induced hyperglycemia [43]. Loss of podocytes in diabetic nephropathy leads to proteinuria and renal failure [44]. We also found evidence of activation of L-selectin ligands by endothelial cells in the glomerular capillary loop of diabetic kidneys suggesting a chronic inflammatory response. Kidneys from D2.*Ins2^Akita/+^* mice showed positive immunoreactivity for the MECA79 antibody, which recognizes the sulfated form of L-selectin ligands in endothelial cells [45]. Sulfation of L-selectin ligands is necessary for the mediation of leukocyte migration to tissues and lymph nodes [45]. In our study, infiltrated leukocytes, positive for CD45, were observed in D2.*Ins2^Akita/+^* glomeruli that were also positive for MECA79. Leukocyte infiltration and activation of inflammatory pathways have also been implicated in the pathology of diabetic nephropathy suggesting a main role of inflammation in renal failure [46]. Although there is no evidence of causative correlation between kidney disease and glaucoma, several genetic disorders such as nail-patella syndrome (LMX1B), oculocerebrorenal syndrome (OCRL) and mutations in the type IV collagen gene (COL4A1), are associated with both development of nephropathy and developmental glaucoma [47], [48], [49], [50]. Due to the premature death of D2.*Ins2^Akita/+^* mice, we were not able to fully explore the impact of severe diabetic nephropathy on DBA/2J glaucoma, although no premature loss of RGCs was observed at 8.5 months of age.

The Akita mutation has previously been shown to cause diabetic retinopathy on a B6 background where loss of RGCs is evident as early as 3 months of age [6], [18], [37]. Contrary to what has been reported in the B6.*Ins2^Akita^* mouse, no loss of RGCs was found in the D2.*Ins2^Akita/+^* at 8.5 months of age, suggesting that the DBA/2J strain is more resistant to diabetes-induced RGC death than the C57BL/6J strain. D2.*Ins2^Akita/+^* do however, show vascular phenotypes commonly associated with diabetic retinopathy, particularly vascular permeability and reactive gliosis. Increased vascular permeability, fluid leakage, deficits in blood flow that lead to hypoxia of retinal tissue, and vascular proliferation are among the most common and diagnosed manifestations of diabetic retinopathy [3], [4]. Although it has been generally thought that neuronal loss in the retina occurs as a consequence of physical breaking of the retinal architecture by proliferative vessels, several studies have shown early loss of RGCs in human and experimental diabetic retinas [5], [6], [7], [8]. Our data, showing vascular leakage but no RGC loss support that at least in some cases, vascular phenotypes precede RGC loss or appear in the absence of RGC loss.

Unfortunately, because of the severe kidney phenotype, we were not able to assess the affects of diabetes on glaucomatous optic neuropathy as this occurs in DBA/2J mice after 9 months of age. However, given that diabetic patients present higher IOP profiles than non-diabetics [9], [10], [11], the D2.*Ins2^Akita/+^* mice do survive long enough to address whether the Akita mutation alters susceptibility to IOP elevation, a major risk factor for glaucoma. Similarly to what has been found in humans, the D2.*Ins2^Akita/+^* mice have a significant increase in IOP at 8.5 months old when compared with wild-type DBA/2J mice. Despite the increase in IOP, coupled with the increase the severity of diabetic nephropathy, in D2.*Ins2^Akita/+^* mice compared to D2.*Ins^+/+^*, no significant differences were detected in ganglion cell loss or in early signs of glaucoma progression including axonal transport. Although several epidemiological studies have suggested diabetes as a risk factor for glaucoma, other studies have not found any association between both diseases [13]. These conflicting results could reside in the different experimental approaches, diagnostics and population used for these studies. Further, these conflicting reports suggest genetic susceptibility plays a major part in disease outcomes, something supported by the differences in both kidney and retinal outcome between the B6 and D2 strains.

We found that chronic diabetes in the DBA/2J mouse increases IOP but does not accelerate the development of early glaucoma in these mice. Also, early signs of glaucomatous RGC dysfunction, such as anterograde axonal transport and axonal degeneration, were not different between diabetic and non-diabetic DBA/2J mice. Therefore, it remains unanswered as to whether diabetes would be an additive stressor to later stage glaucomatous RGC loss in DBA/2J mice or even neuroprotective. A recent epidemiological study suggested that early stages of diabetes could be neuroprotective for glaucoma [14], [15]. Similarly, an experimental study in rat demonstrated neuroprotective effects of diabetes during acute induction of IOP elevation [16]. However, other studies have demonstrated that after optic nerve crush, DBA/2J RGCs are more resistant to RGC death than C57BL/6 due to a genetic locus [51], [52], suggesting that a genetic factor independent of diabetes may be responsible for the strain difference in RGC susceptibility. Identifying such a factor would provide important information for developing drug therapies for treating diabetic complications and RGC loss.
